# Do Young People Perceive E-Cigarettes and Heated Tobacco as Less Harmful Than Traditional Cigarettes? A Survey from Poland

**DOI:** 10.3390/ijerph192214632

**Published:** 2022-11-08

**Authors:** Ilona Wężyk-Caba, Dorota Kaleta, Radosław Zajdel, Łukasz Balwicki, Beata Świątkowska

**Affiliations:** 1 Department of Hygiene and Epidemiology, Medical University of Lodz, 7/9 Żeligowskiego Street, 90-752 Łódź, Poland; 2 Department of Informatics in Business, University of Łódź, POW 3/5 Street, 90-255 Łódź, Poland; 3 Department of Public Health and Social Medicine, Medical University of Gdańsk, 7 Dębinki Street, 80-210 Gdańsk, Poland

**Keywords:** e-cigarettes, heated-tobacco products, harm perception, public health, tobacco, cigarette

## Abstract

New tobacco and nicotine-containing products are gaining more popularity among young people. The aim of this study is to assess the prevalence in the perception of e-cigarettes and heated tobacco among young people in Poland and to assess the factors that are positively correlated with this perception. A cross-sectional study covering almost 12,000 adolescents aged 13–18 was carried out in January and February 2020. Data were collected through a detailed questionnaire recommended for monitoring tobacco use by adolescents. The results of the study showed that 52.2% and 61.9% of young people perceive e-cigarettes and heated tobacco products as less harmful compared to traditional cigarettes, respectively. The risk of perceiving these products as less harmful than smoking was higher among older adolescents, males, those who used these products, had a family member who used e-cigarettes/heated tobacco products and those who were exposed to tobacco advertising. Our study indicates the need to consider the coexistence of traditional smoking, e-cigarettes and heated tobacco and its impact on the assessment of the harmfulness of these products. More research is needed to better understand how perceptions of the harmfulness of e-cigarettes and heated tobacco affect their subsequent use.

## 1. Introduction

New nicotine and other tobacco products, such as electronic cigarettes and heated tobacco products, are new players in the market and consequently a source of scientific interest in the risks associated with these products. In recent years, novel tobacco and nicotine products have been promoted as less harmful and safer than traditional cigarettes and as those that can be helpful in quitting or reducing smoking. Poland abides by the EU Tobacco Products Directive; e-cigarettes are classified as tobacco-related products and vaping is not allowed wherever smoking is prohibited. There is currently no good quality evidence to support the safety hypothesis due to the fact that these products contain potentially harmful chemicals and the addictive component substance, nicotine, is additionally considered to be a tumor promoter [1,2]. The World Health Organization (WHO) shows that evidence of the effectiveness of electronic nicotine delivery systems as methods of smoking cessation are ambiguous and limited, and in particular, that these products are not without harm [3]. Hence, they should be treated with caution as with any other tobacco product, particularly concerning the long-term effects still need to be investigated.

There are concerns as to whether the growing use of e-cigarettes and heated tobacco products in adults increases their attractiveness among young people [4]. Recent published research studies on the perception of e-cigarettes and heated tobacco suggest that respondents believe that e-cigarettes and heated tobacco are less harmful than traditional cigarettes [5,6]. There are also studies that suggest that the percentage of such people is declining and that this type of nicotine product is already seen as harmful [7,8,9].

Perceiving e-cigarettes and heated tobacco as less harmful than traditional cigarettes is important in determining the relationship between this perception and the use of e-cigarettes and heated tobacco. A growing body of research has shown that lower perceptions of the harms of e-cigarettes and heated tobacco are associated with greater popularity and greater likelihood of their use [10,11]. In addition, evidence suggests that there is a significant link between e-cigarette use and future smoking among adolescents who use e-cigarettes [12,13]. The determination of this relationship depending on the current smoking status of traditional cigarettes plays an important role in determining the behavior of young people and can help to understand the mechanisms why young people can be interested in new nicotine and other tobacco products. In addition to assessing this relationship, it is important to identify confounding factors such as parental use of nicotine products, the impact of advertising, or other social factors.

It is interesting to understand how young adults perceive the harmfulness of e-cigarettes and tobacco heated for educational interventions. To our knowledge, published papers on this subject are limited, and therefore the purpose of this work is to assess the prevalence of perceptions of e-cigarettes and heated tobacco by adolescents among a representative sample of young people in Poland and to assess factors that are positively correlated with this perception.

## 2. Materials and Methods

### 2.1. Study Design and Participants Selection

The study was part of a nationwide program for counteracting the health consequences of using tobacco and tobacco products, financed by the National Health Program, the Ministry of Health in Poland. The analysis is based on a large cross-sectional study involving almost 2% of the population of 15–19-year-old primary and secondary school students. The survey was conducted in the first two months of 2020 among 15,225 students from 200 Polish secondary schools on the basis of a random, stratified selection of institutions allowing for 95% confidence in results, 0.5% fraction size and a maximum error of 4%. The presented analysis included 12,618 people who perceived the harmfulness of e-cigarettes and/or heated tobacco.

### 2.2. Methods

The online version of questionnaires, with the prior consent of the participants, were completed using the Computer-Assisted Web Interview tool which increases the reliability of data collection by avoiding errors that may occur during independent data encoding or data entry by using the survey software. The questionnaire was adopted and translated from the Global Youth Tobacco Survey (GYTS) recommended by the World Health Organization (WHO) and the Centers for Disease Control and Prevention (CDC) for monitoring tobacco use by adolescents [14].

Sociodemographic measures included in the analysis gender (boy or girl), age and residence (urban or rural) and type of school (grammar or vocational/technical). Information on smoking status/e-cigarette use/heated tobacco was separately collected. People who never smoked are people who answered “*no*” to the question: “*Have you ever (at least once) used cigarettes*”. Those who answered “*yes*” to this question have been classified as ever smokers. Current smokers were reported to have smoked in the past 30 days. People were also asked about number of e-cigarettes per day.

In order to assess the harmfulness perception of e-cigarettes/heated tobacco products, participants were asked the following separate question: “*How would you rate the harmfulness of e-cigarettes/heated tobacco products compared to regular cigarettes?*” with possible answers: (a) “*Much less harmful*”, (b) “*Less harmful*”, (c) “*Just as harmful*”, (d) “*More harmful*”, (e) “*Much more harmful*” and (f) “*I don’t know/hard to say*”. In our analysis, the responses were divided into much less harmful/less harmful (a and b) compared to others (c–f).

In terms of parental smoking, the respondents were asked about the fact that their parents smoked tobacco and which of the parents smoked: “*Do your parents smoke regular cigarettes?*” with the following answers: (a) “*No, none of the parents smoke cigarettes*”, (b) “*Yes, both my parents smoke cigarettes*”, (c) “*Only daddy smokes*”, (d) “*Only mom smokes cigarettes*” and (e) “*I don’t know*”. Similar questions were asked about e-cigarettes.

For the evaluation of the advertising of nicotine products, respondents were asked about exposure to tobacco marketing in the previous 30 days (conventional cigarettes, e-cigarettes, heated tobacco advertising, offering free tobacco products, offering tobacco products by famous people (e.g., influencers). Those who answered “*yes*” to this question have been classified as those who were exposed to tobacco marketing.

We also asked about the type and intensity of advertising, but these analyzes suggesting insufficient power.

### 2.3. Compliance with Ethics Guidelines

The study was approved by the National Institute of Public Health PZH—Bioethical Committee of the National Research Institute (Resolution No. 3/2019; 13/11/2019).

### 2.4. Statistical Methods

Demographic and clinical characteristics of the study participants, being categorical variables, were presented as frequencies with percentages in brackets. Logistic regression analysis was performed to estimate the odds ratios (ORs) in the logistic regression models. Multivariate logistic regression was adjusted for age and gender and stratified by smoking status. The level of statistical significance was set at 0.05. All statistical analyses were performed using Statistica statistical software (ver.13.0) (StatSoft Poland Inc., Tulsa, OK, USA).

## 3. Results

Socio-demographic and tobacco use characteristics of 12,618 young adult (52.03% men and 47.97% women) are shown in Table 1. The highest proportion of respondents (54.3%) had attained vocational and technical education and the most (77.3%) were averaged 15–17 years. In total, 47.7% of the interviewees resided in rural areas while 53.3% resided in urban areas. The majority of participants were never smokers (43.8%); 29.6% of young people reported current smoking and 26.6% having ever smoked cigarettes but in the past. About 57.8% of individuals reported having used e-cigarettes, including 34.0% current users and 23.8% former users. In total, 51.5% of parents were current smokers and 48.5% were non-smokers. Approximately, 45% of interviewees had noticed some form of tobacco marketing.

Over half of the respondents (52.2%) reported that they believed e-cigarettes were less harmful than conventional cigarettes (CCs). This perception varied by smoking status, with 57.0% of the current smokers, 58.6% of the past smokers and 45.2% of the never smokers perceiving e-cigarettes as less harmful than conventional cigarettes. In total, 61.9% of the interviewees indicated that they believed heated tobacco products (HTPs) to be less harmful than traditional cigarettes, including 69.1% current smokers, 67.9% past smokers and 51.5% never smokers. About 55.97% of respondents reported e-cigarettes and HTPs to be less harmful than CCs. This percentage differed from the smoking status: for current smokers it was 65.5%, for past smokers it was 64.4%, while for never smokers, it was only 42.1% (Table 2).

Table 3 presents results from the multivariate logistic regression, adjusted for age and gender and stratified by smoking status. Female interviewees were consistently less likely to perceive e-cigarettes as less harmful than cigarettes compared to male participants. With regard to smoking status, among current smokers, 92.9%, among past smokers, 84.9%, and among never smokers, 37.9%, reported they never used e-cigarettes. Ever using e-cigarettes was strongly associated with perceiving e-cigarettes as less harmful than conventional cigarettes. Current smokers had 3.43-fold (OR = 3.42; 95% CI: 2.80–4.20) increased risk to perceive e-cigarettes as less harmful than conventional cigarettes compared to smokers who had never used e-cigarettes. E-cigarette users who reported never smoking were more than 3.5 times (OR = 3.52; 95% CI: 3.10–4.01) more likely to perceive e-cigarettes as less harmful than traditional cigarettes compared to never smokers without a history of e-cigarette use. In addition, past smokers were just over three times (OR = 3.11; 95% CI: 2.62–3.67) more likely to perceive e-cigarettes as less harmful than conventional cigarette smoking compared to past smokers who had never tried e-cigarettes. The number of e-cigarettes per day showed a dose–response trend toward increasing the risk in perception of e-cigarettes as less harmful than cigarettes among current smokers. A higher number of e-cigarettes was associated with a higher risk for this perception (>15 e-cigarettes per day: OR = 5.52; 95% CI: 3.92–7.78).

About 44% of respondents reported having used heated tobacco products. With regard to smoking status, among current smokers, 69.0%, among past smokers, 45.0% and among never smokers, 12.6% reported they were never users of heated tobacco products. Similar to e-cigarettes cases, ever use heated tobacco product was related to the risk in perceptions about the health risks associated with these products as being less harmful compared to traditional cigarettes. Current cigarette smokers were almost 2.5 times more likely to perceive heated tobacco products as less harmful than conventional cigarettes (OR = 2.44, 95% CI: 2.05–2.91), odds for past smokers OR = 2.92 (95% CI: 2.35–3.63) and lastly never cigarette smoking status was related to an almost five times greater risk in perceiving heated tobacco products as less harmful than conventional tobacco products (OR = 4.94; 95% CI: 3.47–7.04) (Table 4). Taking into account the intensity of smoking of heated tobacco products, the relationship between their greater use and the increased risk of considering these products as less harmful than traditional smoking products was in the group of current smokers. The highest risk was observed in the last group (>15 HTPs per day) and was OR = 2.74; 95% CI: 1.69–4.43.

Parental e-cigarette use was a significant determinant in the risk of perceiving e-cigarettes as less harmful than tobacco smoking. Among current smokers, 9.4% had a history of parents’ e-cigarette use, and these smokers were more likely to perceive e-cigarettes as less harmful than conventional tobacco cigarettes (OR = 1.37; 95% CI: 1.06–13.77) compared to smokers whose parents did not use e-cigarettes. The risk increased to OR = 1.58 (95% CI: 1.20–2.10) and to OR = 2.29 (95% CI: 1.64 = 3.20) in adolescents who reported that their parents were not e-smokers and who never smoked and among past smokers, respectively. In addition, analysis showed that young men of parents who e-smoked had the higher risk of perceiving e-cigarettes as less harmful than traditional smoking. Father-only e-cigarette use was associated with OR = 2.29 (95% CI: 1.32–3.99) among current smokers, OR = 3.97 (95% CI: 2.01–7.85) among past smokers and with a risk of 3.15 if they had never smoked. The risk of perceiving e-cigarettes as less harmful than traditional cigarettes in teenagers who had never smoked were more than four times higher (OR = 4.16; 95% CI: 1.14–15.12) when both parents were e-smokers. With regard to heated tobacco products, such analyses suggests insufficient power.

Exposure to tobacco marketing was associated with a higher risk of perceiving e-cigarettes as less harmful than conventional cigarettes, but not for current smokers (OR = 1.03; 95% CI: 0.89–1.19). These relationships varied by smoking status and were similar for past and non-smokers, OR = 1.23 (95% CI: 1.05–1.43) and 1.29 (95% CI: 1.15–1.46), respectively. The analysis showed a similar relationship for exposure to tobacco marketing and perceiving heated tobacco smoking as less harmful than conventional cigarettes (OR = 1.43; 95% CI: 1.16–1.77 for current smokers, OR = 1.88; 95% CI: 1.47–2.40 for past smokers and 1.47; 95% CI:1.19–1.81 among never smokers).

## 4. Discussion

The results of the study showed that 52.2% (more than half) and 61.9% of young people perceive e-products and heated tobacco as less harmful compared to traditional cigarettes. We also found that the perception of e-cigarettes as not harmful was lower among young people who had never smoked traditional cigarettes (45.2%). Regarding young people who use heated tobacco, the highest percentage of those who rated these products as less harmful was among current tobacco smokers (69.1%). The perception of e-cigarettes and heated tobacco products as less harmful than traditional cigarettes, established in our study, is in line with previous research on the perception of other nicotine and tobacco products among young adults [15,16,17,18,19,20].

The risk of perceiving these products as less harmful than traditional smokers was higher among older adolescents, males, those who used these products, had a family member who used e-cigarettes/heated tobacco products and those who were exposed to tobacco advertising. A similar relationship was observed, with the exception of parental smoking, where the variable was not significantly correlated for heated tobacco. Interestingly, our study found that having ever used e-cigarettes is related to perceiving e-cigarettes as less harmful than traditional cigarettes, hence it may be a predictor of more frequent use of e-cigarettes. This explanation is consistent with previous research [21,22].

With regard to social factors, the study found that family e-cigarette use was linked to perception of e-cigarettes. Risk of perceiving e-cigarettes as less harmful than traditional cigarettes when both parents were e-smokers was more than four times higher (OR = 4.16; 95% CI: 1.14–15.12) than in adolescents without a history of smoking by their parents. This may suggest that parental e-cigarette use may increase the likelihood of young people using e-cigarettes. However, this was not the aim of the study, but if this perception was related to the subsequent use of these products, it would be a worrying result, especially in the non-smoking group.

The advertising was also related to the perception of the harmfulness of e-cigarettes and heated tobacco products. Scientific evidence of a link between exposure to tobacco advertising and increased use of tobacco, including e-cigarettes, has been implicated before. Published data show that e-cigarette marketing may have an impact on enabling potential users to perceive e-cigarettes as being safer for conventional cigarettes [23,24,25,26,27]; however, more detailed research could verify this thesis and determine whether the advertisement influences the perception in harmfulness of new nicotine and other tobacco products.

Although the conducted research provides interesting information on the perception of harmfulness of e-cigarettes and smoked tobacco among young people and related factors, it also has some limitations. First, the data obtained are cross-sectional and therefore it is not possible to investigate the relationship between the perception of harmfulness and the subsequent likelihood of using these products in the future. Additionally, these preferences are likely to change over time. Secondly, although the survey is representative, it concerns the population of young Poles, where the problem of e-cigarettes and heated tobacco has appeared relatively recently. One limitation of the study is also the lack of an assessment of nicotine perception in e-cigarettes and heated tobacco products, which may differ from the assessment on the perception of these products. It is likely that young people may experiment with e-cigarettes without fully understanding the role of nicotine in some products, including e-cigarettes and heat-treated tobacco. This limitation in our study was minimized by grouping respondents according to the smoking status of traditional cigarettes. Hence, in the future, research is needed on the nicotine content in e-cigarettes and their relationship to the knowledge on this subject among young people, and thus a better understanding of the results obtained.

Despite the indicated limitations, the results of our analysis have important advantages, for example, the assessment is disseminated by the perception of e-cigarettes and heated tobacco as less harmful in the population of young adults and the social factors associated with this relationship.

## 5. Conclusions

Understanding how young adults perceive the harmfulness of e-cigarettes and heated tobacco can help in educational interventions that address the use of new forms of nicotine and other tobacco products.

Educational programs should inform people that while e-cigarettes typically contain less chemicals than conventional cigarettes, e-cigarettes and aerosols are not harmless. It is important to underline the lack of clear results and uncertainty about the long-term health consequences of e-cigarette and heated tobacco use. The results of our study also show the need for an interest in the coexistence of traditional cigarettes and e-cigarettes and the associated perception of harmfulness in preventive interventions related to smoking cessation among young people.

In conclusion, more research is needed to better understand how the perception of the harmfulness of e-cigarettes and heated tobacco influences subsequent use of these products, especially in non-smokers, and what determines the change in the use of nicotine products among both smokers and never-smokers.

## Figures and Tables

**Table 1:** Characteristics of respondents.

Variables	Total n (%)
Population (overall)	12,618 (100.00)
Age (years)	
15–17	9752 (77.29)
≥18	2866 (22.71)
Type of school	
grammar	5731 (45.65)
vocational/technical	6824 (54.35)
Place of residence (number of inhabitants)	
rural	5818 (47.68)
cities < 20th	2549 (20.89)
cities 20–99th	2147 (17.60)
cities > 100th	1688 (14.82)
Smoking (traditional cigarettes)	
never	5527 (43.80)
past	3359 (26.62)
current	3732 (29.58)
Smoking (e-cigarettes)	
never	5322 (42.18)
past	2999 (23.77)
current	4297 (34.05)
Parental smoking	
no	5771 (48.48)
yes	6133 (51.52)
traditional cigarettes	5063 (42.42)
e-cigarettes	685 (5.81)
heated tobacco	385 (3.33)
Exposure to tobacco marketing	
no	6398 (54.89)
yes	5259 (45.11)

**Table 2:** Perception of e-cigarettes and heated tobacco by conventional cigarette smoking status.

Variables	Total n (%)	Current Smokers n (%)	Past Smokers n (%)	Never Smokers n (%)
E-cigarettes harm perception class				
similar/more harmful/far more harmful	6028 (47.77)	1606 (43.03)	1392 (41.44)	3030 (54.82)
far less harmful/less harmful	6590 (52.23)	2126 (56.97)	1967 (58.56)	2497 (45.18)
Heated tobacco harm perception class				
similar/more harmful/far more harmful	2778 (38.08)	771 (30.89)	631 (32.13)	1376 (48.52)
far less harmful/less harmful	4518 (61.92)	1725 (69.11)	1333 (67.87)	1460 (51.48)
E-cigarettes and heated tobacco harm perception class				
similar/more harmful/far more harmful	2089 (44.03)	551 (34.48)	452 (35.56)	1086 (57.92)
far less harmful/less harmful	2655 (55.97)	1047 (65.52)	819 (64.44)	789 (42.08)

**Table 3:** Factors associated with perceiving e-cigarettes as less harmful than traditional cigarettes, by cigarette smoking status.

Variables	Current Smokers (%)			Past Smokers (%)			Never Smokers (%)		
	n (%)	OR	95% CI	n (%)	OR	95% CI	n (%)	OR	95% CI
Gender									
male	1321 (62.14)	1.00	—	1241 (63.14)	1.00	—	1554 (62.23)	1.00	—
female	805 (37.86)	0.45	0.39–0.51	725 (36.86)	0.33	0.29–0.38	944 (37.77)	0.43	0.39–0.48
Ever uses of e-cigarettes	1973 (92.80)	3.43 *	2.79–4.19	1670 (84.90)	3.11 **	2.64–3.67	946 (37.89)	3.52 ***	3.10–4.01
Number of e-cigarettes per day									
never	261 (17.33)	1.00	—	763 (56.64)	1.00	—	588 (71.27)	1.00	—
1	232 (15.40)	2.58	1.99–3.58	122 (9.06)	2.89	1.97–4.24	72 (8.73)	3.63	1.98–6.64
2–4	278 (18.46)	3.56	2.73–4.65	142 (10.54)	3.66	2.48–5.40	55 (6.67)	4.00	1.69–8.19
5–9	257 (17.07)	4.67	3.49–6.25	135 (10.0)	4.74	3.05–7.35	38 (4.46)	4.98	1.94–12.75
10–15	286 (18.99)	5.07	3.80–6.76	125 (9.28)	5.77	3.52–9.45	50 (6.06)	2.52	1.35–4.70
>15	192 (12.75)	5.52	3.93–7.78	60 (4.45)	3.29	1.88–5.76	22 (2.67)	—	—
Parental e-cigarettes use									
no	1753 (90.64)	1.00	—	1679 (62.10)	1.00	—	2247 (95.01)	1.00	—
yes	181 (9.36)	1.37	1.06–13.77	144 (7.90)	2.29	1.64–3.20	118 (4.99)	1.58	1.20–2.10
son whose mother uses e-cigarettes	35 (1.81)	2.43	1.23–4.81	19 (1.04)	2.42	0.96–6.08	16 (0.68)	1.25	0.62–2.5
son whose father uses e-cigarettes	51 (2.64)	2.30	1.13–3.99	52 (2.85)	3.98	2.02–7.85	48 (2.03)	3.15	1.85–5.37
son whose both parents uses e-cigarettes	33 (1.71)	1.40	0.79–2.50	15 (0.82)	2.87	0.98–8.66	10 (0.42)	4.16	1.14–15.12
Exposure to tobacco marketing									
no	817 (43.39)	1.00	—	904 (50.08)	1.00	—	1395 (59.04)	1.00	—
yes	1066 (56.61)	1.03	0.89–1.19	901 (49.92)	1.23	1.05–1.43	968 (40.96)	1.30	1.15–1.46

* Compared to smokers who do not use e-cigarettes; ** compared to ex-smokers who do not use e-cigarettes; *** compared to non-smokers who do not use e-cigarettes.

**Table 4:** Factors associated with perceiving heated tobacco products as less harmful than traditional cigarettes, by cigarette smoking status.

Variables	Current Smokers (%)			Past Smokers (%)			Never Smokers (%)		
	n (%)	OR	95% CI	n (%)	OR	95% CI	n (%)	OR	95% CI
Gender									
male	993 (57.57)	1.00	—	789 (59.19)	1.00	—	880 (60.27)	1.00	—
female	732 (42.43)	0.82	0.67–0.98	544 (40.81)	0.57	0.47–0.69	580 (39.73)	0.67	0.58–0.78
Ever used heated tobacco products	1204 (69.80)	2.44 *	2.05–2.91	600 (45.01)	2.92 **	2.35–3.63	184 (12.60)	4.94 ***	3.47–7.04
Number of heated tobacco products									
never	113 (14.79)	1.00	—	303 (51.53)	1.00	—	182 (63.64)	1.00	—
1	116 (15.18)	1.71	1.12–2.62	53 (9.01)	1.93	1.03–3.26	30 (10.49)	2.72	1.21–6.12
2–4	143 (18.72)	2.11	1.39–3.20	63 (10.71)	2.64	1.44–4.86	27 (9.44)	9.79	2.29–41.90
5–9	133 (17.41)	2.25	1.46–3.47	70 (11.90)	3.43	1.81–6.50	15 (5.24)	3.62	1.03–12.78
10–15	149 (19.50)	2.58	1.68–3.97	65 (11.05)	2.45	1.28–3.95	21 (7.34)	2.54	0.99–6.46
>15	110 (14.40)	2.74	1.69–4.43	34 (5.78)	3.99	1.53–10.40	11 (3.85)	3.99	0.87–18.30
Exposure to tobacco marketing									
no	361 (38.24)	1.00	—	341 (45.47)	1.00	—	409 (54.17)	1.00	—
yes	583 (61.76)	1.43	1.16–1.77	406 (54.53)	1.88	1.47–2.40	346 (45.83)	1.47	1.19–1.81

* Compared to smokers who do not use heated tobacco products; ** compared to ex-smokers who do not use heated tobacco products; *** compared to non-smokers who do not use heated tobacco products.

## Data Availability

Available online: https://www.pzh.gov.pl/wp-content/uploads/2020/06/RAPORT-TYTOŃ-MŁODZIEŻ-GRUDZIEŃ-2019-WERSJA-FINALNA-www.pdf (accessed on 13 September 2022).
